# Tea-Soybean Intercropping Improves Tea Quality and Nutrition Uptake by Inducing Changes of Rhizosphere Bacterial Communities

**DOI:** 10.3390/microorganisms10112149

**Published:** 2022-10-29

**Authors:** Litao Sun, Xue Dong, Yu Wang, Garth Maker, Manjree Agarwal, Zhaotang Ding

**Affiliations:** 1 Tea Research Institute, Shandong Academy of Agricultural Sciences, Jinan 250100, China; 2 College of Science, Health, Engineering and Education, Murdoch University, 90 South Street, Perth, WA 6150, Australia; 3 College of Food Science and Engineering, Nanjing University of Finance and Economics, Nanjing 210023, China; 4 Tea Research Institute, Qingdao Agricultural University, Qingdao 266109, China; 5 Scientific Service Division, Chemcentre, Government of Western Australia, B.No. 500, Corner of Manning Road and Townsing Drive, Bentley, WA 6102, Australia

**Keywords:** tea/soybean intercropping, catechins, tea quality, bacterial community, soil fertility

## Abstract

The positive aspects of the tea plant/legume intercropping system draw attention to the Chinese tea industry for its benefit for soil fertility improvement with low fertilizer input. However, limited information exists as to the roles of intercropped legumes in the rhizosphere microbiome and tea quality. Hereby, soybean was selected as the intercropped plant to investigate its effect on bacterial communities, nutrient competition, tea plant development, and tea quality. Our data showed that intercropped soybean boosted the uptake of nitrogen in tea plants and enhanced the growth of young tea shoots. Nutrient competition for phosphorus and potassium in soil existed between soybeans and tea plants. Moreover, tea/soybean intercropping improved tea quality, manifested by a significantly increased content of non-ester type catechins (C, EGC, EC), total catechins and theanine, and decreased content of ester type catechins (EGCG). Significant differences in rhizobacterial composition were also observed under different systems. At the genus level, the relative abundance of beneficial bacteria, such as *Bradyrhizobium, Saccharimonadales* and *Mycobacterium,* was significantly increased with the intercropping system, while the relative abundance of denitrifying bacteria, *Pseudogulbenkiania,* was markedly decreased. Correlation analysis showed that *Pseudogulbenkiania, SBR1031,* and *Burkholderiaceae* clustered together showing a similar correlation with soil physicochemical and tea quality characteristics; however, other differential bacteria showed the opposite pattern. In conclusion, tea/soybean intercropping improves tea quality and nutrition uptake by increasing the relative abundance of beneficial rhizosphere bacteria and decreasing denitrifying bacteria. This study strengthens our understanding of how intercropping system regulate the soil bacterial community to maintain the health of soils in tea plantations and provides the basis for replacing chemical fertilizers and improving the ecosystem in tea plantations.

## 1. Introduction

The tea plant (*Camellia sinensis* (L.) O. Kuntze) is widely cultivated as an economic crop in tropical and subtropical areas. In 2020, the global cultivated area of tea was 5.31 million hectares, and 7.02 million tonnes was produced across 47 countries [1]. China has the most land devoted to tea production, at 3.37 million hectares, accounting for ~63.4% of the global tea harvest area. As the largest tea producer, China produced around 2.98 million tonnes of tea, making up 42.5% of global production [1]. Tea plants require low pH soil; however, soil acidification and soil fertility degradation occur frequently in tea plantations [2], attributed to non-ecofriendly management, such as long-term and substantial application of chemical fertilizer [3,4].

Intercropping, an eco-friendly agricultural practice of growing two or more crops together [5], not only alleviates soil acidification and improves soil fertility, enabling improved crop yield and quality [6,7], but also substantially optimizes and diversifies cropping systems. Intercropping of tea plants with legumes has been applied in many plantations, reducing inter-specific competition by enhancing complementarity/facilitation processes, thereby increasing tea yield [8], changing physicochemical composition of tea leaves [9], and improving nutrient availability and enzyme activity in the soil [10].

In addition, underground interactions between intercropped roots and soil organisms are drawing specific attention. Recent research into nutrient-utilization in cereal/legume, cassava/peanut, and sugarcane/soybean intercropping suggested the critical role of soil bacterial diversity in the rhizosphere in these processes [11,12,13]. Several theories have been proposed to explain the effect of legume intercropping on the structure of rhizobacterial communities. It has been demonstrated that legume secretion such as flavonoids, have the ability to influence plant growth-promoting rhizobacteria (PGPR) mobility, improving root colonization and phytobeneficial activity of these PGPR, leading to plant facilitation [14,15]. Another example is that N-acyl homoserine lactone (N-AHL) in several legume root exudates affects gene expression in quorum sensing, a bacterial regulation process [16,17]. Bacterial activity is also influenced by intercropping legumes via a priming effect [18]. An increase in soil organic matter (SOM) mineralization by adding fresh organic matter from legumes was observed, stimulating the activity of soil bacteria communities involved in the mineralization of stable SOM [19,20].

Soil bacterial diversity is sensitive to cultivation methods, which affect soil physicochemical properties. Previous research reported that the most dominant bacterial phyla in tea plantations are Acidobacteria, Proteobacteria, Chloroflexi, Actinobacteria, and Firmicutes [21,22]. Li, et al. [23] indicated that continuous (10- and 20-year) tea cultivation altered the soil bacterial community, resulting in the reduction in the relative abundance of some beneficial bacteria, such as *Pseudomonas, Rhodanobacter, Bradyrhizobium, Mycobacterium, and Sphingomonas*. It has been demonstrated that long-term tea monoculture adversely affected soil bacterial diversity [23,24], while long-term nitrogen fertilization impeded recruitment of PGPR, hampering beneficial interactions between microbe and plant host [25]. Intercropping can affect the diversity and composition of the soil bacterial community in tea cultivation. Legume/tea intercropping was observed to increase the relative abundance of beneficial bacteria including *Acidobacteriaceae, Burkholderiaceae, Rhodanobacteraceae,* and *Sphingomonadaceae,* which are considered to be organic matter decomposers or PGPR [26]. Additionally, Huang, et al. [27] found that legume/tea intercropping significantly increased absolute abundance of *Bacillus,* which are defined as PGPR contributing to promotion of crop production [28].

While some studies have analyzed the effect of intercropping on bacterial composition in tea plantations, the variation, abundance, and function of bacterial communities in the tea garden intercropping system remain to be explored. Discrepancies have been observed in the effect of intercropping systems on the soil bacterial community, mainly caused by crop species, tree age, and even growth stage of crops [29]. A previous study indicated that metabolites in tea plants such as flavonoids, amino acids, and carbohydrates significantly changed when intercropping with soybean growth to the profuse flowering, promoting tea growth and improving tea quality [9]. However, variation in the rhizosphere bacterial community during the legume profuse flowering stage, and the resulting influence on tea quality and development are still unknown. This study aims to investigate the impacts of intercropping on soil fertility and tea plant performance and to characterize rhizosphere bacterial community composition during the legume profuse flowering stage by 16S rRNA gene amplicon sequencing.

## 2. Materials and Methods

### 2.1. Tea-Soybean Intercropping Experiment Design

The pot experiment was conducted in the phytotron at Qingdao Agricultural University. Conditions were as follows: temperature, 25 °C/18 °C (16 h light/8 h dark); humidity, 75%; and light intensity, 18,000 Lx. Fresh soil was collected from the surface layer (0~20 cm) of a tea plantation and was passed through a 5 mm mesh size sieve. The soil was classified as brown loamy soil with major chemical characteristics as follows: pH 5.6, organic matter (OM), 16.85 g/kg, total nitrogen (TN), 1.18 g/kg, total phosphorus (TP), 1.18 g/kg, total potassium (TK), 1.18 g/kg, available nitrogen, 106.08 mg/kg, available phosphorus, 47.76 mg/kg and available potassium, 160.27 mg/kg. One-year-old *Camellia sinensis* (L.) O. Kuntze “Zhongcha 108” and *Glycine max* (L.) merrill “HuangChun 2” were selected for cultivation. Tea plants with the same growth conditions were chosen as candidates for further experiments. A randomized complete block design with tea plant monoculture (control, CK) and tea plant intercropping with soybean was performed. Each treatment contained thirty plastic pots (93 × 80 × 100 mm).

### 2.2. Plant and Soil Sample Collection and Physicochemical Analysis

Tea shoots (a bud and two expanding leaves) and tender roots were collected from each treatment when the intercropped soybean plants were profusely flowering (three biological replicates). The 2,3,5-triphenyltetrazolium chloride (TTC) method was used to determine root activity [30]. Whole tea plants and soybeans were oven dried at 60 °C for 48 h, ground, and meshed through 0.2 mm for TN, TP, and TK at the Soil Testing Laboratory. In the same period, soils tightly attached to the root surface (0~5 mm) was collected after vigorous shaking. Each sample was a pool of three randomly selected plants and was homogenized by mixing through a 2-mm sieve to discard plant residue. Each soil sample was divided into two parts: one part was snap frozen in liquid nitrogen and stored at −80 °C until soil DNA extraction; the other was air dried at room temperature, ground, and meshed through 0.2 mm for TN, TP, and TK at the Soil Testing Laboratory.

### 2.3. Competitive Ratio between Tea Plants and Soybeans in the Intercropping System

Competitive ratio (CR) of N, P, and K was employed to assess the competition intensity between tea plants and soybeans [31,32]. It is calculated as follows:

$$XCR=\frac{X_{it}}{X_{mt}\times F_{t}}÷\frac{X_{is}}{X_{ms}\times F_{s}}$$

where *XCR* is the competitive ratio of N, P, and K in the tea plant–soybean intercropping system; *X_it_*, nutrient (TN, TP, and TK) content per unit area of tea plants grown in the intercropping system; *X_mt_*, nutrient (TN, TP and TK) content per unit area of tea plants grown in monoculture; *X_is_*, nutrient (TN, TP and TK) content per unit area of soybeans grown in the intercropping system; *X_ms_*, nutrient (TN, TP, and TK) content per unit area of soybeans grown in monoculture; *F_t_* and *F_s_*, the proportion of intercropped area occupied by tea plants and soybeans in the intercropping system, respectively. In this experiment, the cultivation area ratio between tea plants and soybeans was considered as 1:1. Tea plants are more competitive than soybeans if CR_ts_ > 1 and vice versa.

### 2.4. DNA Extraction and 16S rRNA Gene Sequencing

The extraction of soil DNA was followed by our previously described method [33,34]: E.Z.N.A.^®^ Soil DNA Kit (Omega Bio-tek, Norcross, GA, USA) was applied to extract microbial DNA, according to manufacturer’s instructions. NanoDrop 2000 ultraviolet-visible spectrophotometer (Thermo Scientific, Wilmington, NC, USA) was used to determine the final DNA concentration and purified concentration, and the DNA quality was checked by 1% agarose gel electrophoresis. V3–V4 hypervariable regions were amplified using forward primers 338F (5′-ACTCCTACGGGAGGCAGCAG-3′) and 806R (5′-GGACTACHVGGGTWTCTAAT-3′) for 16S rRNA gene in bacteria. The procedure used in the PCR reaction is described in a previous study [33]. Purified amplicons of equal molecular weight were pooled on the Illumina MiSeq platform (Illumina, San Diego, CA, USA) and subjected to paired-end sequencing (2 × 300) according to the standard protocol of MajorbioBio-Pharm Technology Co., Ltd. (Shanghai, China).

The data were analyzed on the online platform of Majorbio Cloud Platform (www.majorbio.com; accessed on 20 July 2022). UPARSE (version 7.1; http://drive5.com/uparse/; accessed on 1 August 2022) was employed to pre-clustered operational taxonomic units (OTUs) at 97% similarity. OTUs were subsequently mapped to the Silva database (Release132; http://www.arb-silva.de; accessed on 20 August 2022) through the RDP classifier algorithm (http://rdp.cme.msu.edu/; accessed on 20 August 2022). Raw sequencing data were deposited in NCBI Sequence Read Archive (SRA) and the range of 9 SRA accession numbers were SRX17146438–SRX17146446.

### 2.5. Quantitative Determination of Tea Quality Components

The content of catechins, caffeine, and theanine were quantified as described in our previous study [35,36]. For catechins and caffeine, approximately 0.4 g of fresh tea leaves were ground to a fine powder with liquid nitrogen, followed by sonication by extraction with 5 mL extraction solution (80% methanol, 20% water), and centrifugation at a relative centrifugal force (RCF) of 4000× *g* for 15 min. The residues were re-extracted twice by the same procedure. All steps were performed at a low temperature (~0 °C). Chloroform was employed to extract supernatants, three times. Before high-performance liquid chromatography (HPLC) analysis, samples were filtered through a 0.22 µm polyethersulfone membrane. The mobile phase was (A) double distilled water/acetonitrile/acetic acid/EDTA (88.8/9/2/0.2; *v/v/v/v*) and (B) double distilled water/acetonitrile/acetic acid/EDTA (17.8/80/2/0.2; *v/v/v/v*). The samples were eluted at a flow rate of 1 mL/min, with three independent extractions. HPLC-grade standard chemicals, including epicatechin (EC), epigallocatechin (EGC), catechin (C), epicatechin gallate (ECG), epigallocatechin gallate (EGCG), caffeine and theanine were purchased from Sigma Aldrich (St. Louis, MO, USA).

For theanine analysis, approximately 1 g of fresh tea leaves was ground to a fine powder with liquid nitrogen, followed by brewing with 100 mL boiling distilled water for 10 min. Cooled extracts were filtered through a 0.45 µm nylon membrane, following 10 min centrifugation at 13,000 r/min. The mobile phase of HPLC was water (A) and acetonitrile (B).

### 2.6. Statistical Analysis

The difference between two groups was calculated and statistically examined by independent-sample t-tests (SPSS, Inc., Chicago, IL, USA). To investigate the patterns of separation between microbial communities, principal coordinate analysis (PCoA) calculated with Phyloseq package (v.1.10) was used, based on the Bray–Curtis distances. The composition of the bacterial community was analyzed by linear discriminant analysis (LDA) coupled with effect size measurements (LEfSe) (http://huttenhower.sph.harvard.edu/galaxy/; accessed on 1 September 2022). Welch’s t-test was applied to identify phyla and genera that showed significant differences in abundance between groups (confidence interval method). Correlation of rhizosphere soil bacteria-physicochemical was calculated by performing Spearman’s rank correlation analysis and a heat map was drawn using “pheatmap” package in R software.

## 3. Results and Discussion

### 3.1. Effect of Tea Plant/Soybean Intercropping on Tea Plant Development

**Figure 1:**
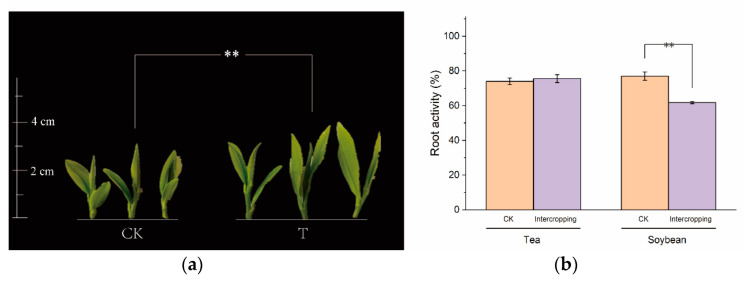
Effect of tea plant/soybean intercropping on (**a**) growth performance and (**b**) root activity. CK: tea plant in monoculture. T: tea plant in tea plant/soybean intercropping system. All data are shown as the mean ± SD (*n* = 3). Asterisks represent significant differences (Tukey’s HSD test, *p* < 0.05).

Intercropping with legumes is considered as a productive and sustainable system. A growing emphasis is being placed on the positive aspects of the tea plant/soybean intercropping system in Chinese tea industry [4,37]. This aligns with the development philosophy of achieving carbon peaking and carbon neutrality goals. Previous studies revealed that the intercropped soybean, especially at the profuse flowering stage, can significantly affect the synthesis and metabolism of amino acids, further improving tea quality. In this study, the effect of the soybean intercropping system at the profuse flowering stage on the tea plant growth was investigated. Young tea shoots (a bud and two expanding leaves) and tender tea roots were collected when the intercropped soybean plants were profusely flowering. It was observed that the development of young tea shoots markedly improved in the tea plant/soybean intercropping system compared with CK (tea plants under monoculture system) (Figure 1). These observations are in accordance with Ma, et al. [38]. Duan, et al. [9] revealed that intercropping soybeans were beneficial to the growth and development of tea plants by increasing stomatal conductance (Gs), net photosynthetic rate (Pn) and transpiration rate (Tr) of the intercropping tea plant. With regard to root activity, there were no significant differences between the tea plants in different culture systems, but the root activity of soybean reduced from 77.00% to 61.70%, indicating interspecies competition between tea plants and soybean. This could be due to the changed nutrient conditions and microenvironment induced by the intercropped tea plants.

### 3.2. Effect of Tea Plant/Soybean Intercropping on N, P and K

N, P, K are essential nutrients for tea to maintain growth and quality. Soybean intercropping increases soil fertility with low fertilizer input. Many studies demonstrated that, compared with the other plants, legumes were less competitive in absorbing nitrogen from the soil, and their root nodules fixed nitrogen up to 15% under a legume-crop intercropping system, thereby reducing inorganic fertilizer requirement [39,40]. To evaluate variation of nutrients in soil under tea plant/soybean intercropping, the content of N, P, and K in soil, soybean, and tea plants were analyzed (Table 1). There were no significant differences in N and K between monoculture and intercropping soil, but P levels were higher in the tea plant monoculture soil than intercropping soil, consistent with the suggestion that soil-available P is much lower under a soybean/tea intercropping system [30].

Regarding nutrients in plants, N markedly increased in both tea and soybean plants for intercropping, in agreement with Duan et al. [30] who suggested that N in soybean/tea-intercropped tea plants was increased compared to that in monoculture, as legumes can improve soil-available nitrogen by biological nitrogen fixation by root-nodule bacteria. Nitrogen is essential for forming caffeine, amino acids, and other chemical ingredients of tea leaves. However, TP in soybean increased and that in tea plants decreased when subjected to intercropping treatment. This could be attributed to the different interspeciescompetition between intercropped tea plants and soybean. For each plant, K content showed no significant difference between monoculture and intercroppingsystems. Additionally, K content in soybean was higher than that in tea plants no matter under monoculture or intercropping system, which suggested that intercropping could not change the distribution of K resource between tea and soybean in this study. However, there was less information on the reason that soybean could acquire more K than tea plant.

**Figure 2:**
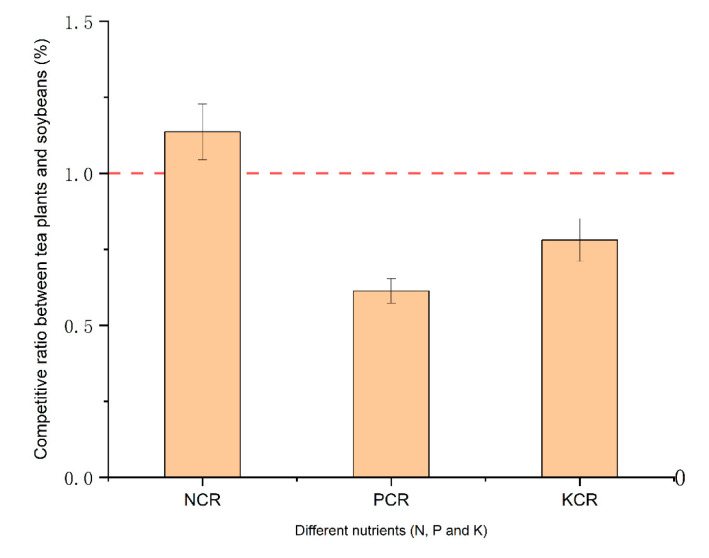
Competitive ratio for nitrogen, phosphorus and potassium in the tea plant/soybean intercropping system. The dashed line indicates the mean (*n* = 3) value of XCR = 1.

Subsequently, the competitive ratio (CR) was employed to assess competition intensity for nutrients between tea plants and soybeans. It evaluated the performance of intercropping to decide whether the intercropped soybeans provided benefit to the tea plants based on the nutrient competition. As shown in Figure 2, the nitrogen competitive ratio (NCR) of tea plants was higher than that of soybeans, whereas the phosphorus (PCR) and potassium (KCR) competitive ratios of tea plants were lower than that of soybeans. This indicated that the nitrogen fixation capacity of legumes relieved competition for nitrogen in the tea plant–soybean intercropping system, while the soybean still required P and K from the soil, which led to nutrition competition. Compared with the other intercropped non-legume plants, the N_2_-fixing bacteria in soybean can provide N by biological nitrogen fixation [41].

### 3.3. Quality-Related Components in Tea Leaves under Tea Plant-Soybean Intercropping

**Figure 3:**
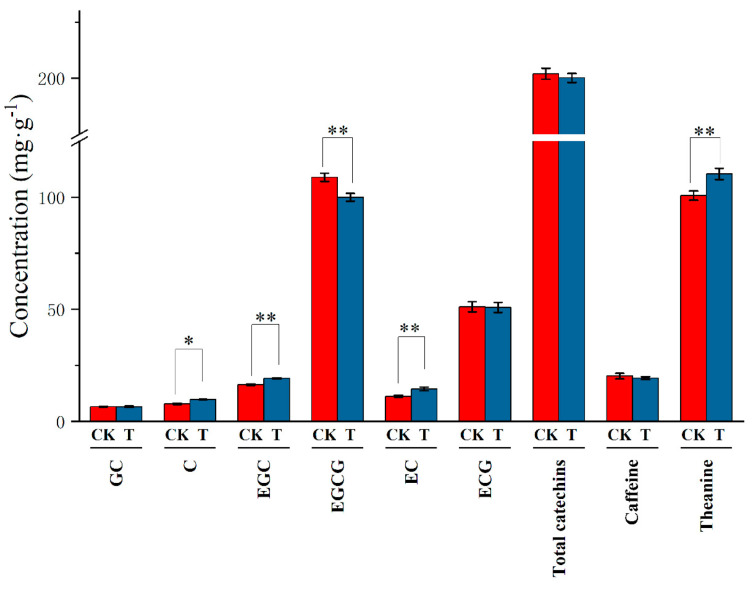
Change in quality-related components in tea leaves under tea plant–soybean intercropping. CK: tea plant in monoculture. T: tea plant in tea plant/soybean intercropping system. All data are shown as the mean ± SD (*n* = 3). Asterisks represent significant differences (Tukey’s HSD test, * represents *p* < 0.05, ** represents *p* < 0.01).

Catechins, amino acids, and caffeine are considered as the three main components of tea quality [42], with catechins being the key components, as they impart the taste of astringency and bitterness of the final tea infusion. Catechins can be divided into two groups, nonester type catechins (gallocatechin, GC; catechin, C; epigallocatechin, EGC; epicatechin, EC) and ester type catechins (simple catechins) (epigallocatechin gallate, EGCG; epicatechin gallate, ECG). Ester type catechins are the main contributors to the characteristic astringency and bitterness of tea, whereas simple catechins are of slight astringency, and therefore more beneficial to tea quality [43]. As shown in Figure 3, the content of C, EGC, EC, and total catechins in the tea leaves under the intercropping system was greater than in the monoculture system. In contrast, the content of EGCG was significantly decreased under the intercropping system. It indicated that intercropping tea plants with soybean can enhance the flavor of the tea infusion without increasing the astringency and bitterness.

Amino acids, particularly theanine, impart the characteristic umami of tea infusion [44]. It was observed that the tea–soybean intercropping system significantly increased the content of theanine in the young tea leaves. Duan, et al. [9] also reported that intercropping with soybean could promote amino acid metabolism. One reason could be that soybean increased N availability in the soil by nitrogen fixation through root-nodule bacteria, which benefits nitrogen absorption of the tea plant and provides the basic N source for the synthesis of amino acids. Another reason could be that intercropping with soybean could create a relatively stable niche by enhancing facilitative roots and microbial processes, which could benefit the amino acid biosynthesis.

No significant effect of soybean intercropping on the content of caffeine in tea leaves was observed. Huang, et al. [27] conducted field research and reported that the content of caffeine was significantly lower under intercropping with legumes than under monoculture. However, Duan, et al. [9] reported that no significant difference in caffeine content was found between monoculture and intercropping systems, which is consistent with our results. Hence, more factors need to be considered in the synthesis of caffeine in tea leaves.

### 3.4. Diversity of the Bacterial Community in Rhizosphere Soil under Tea Plant-Soybean Intercropping

**Figure 4:**
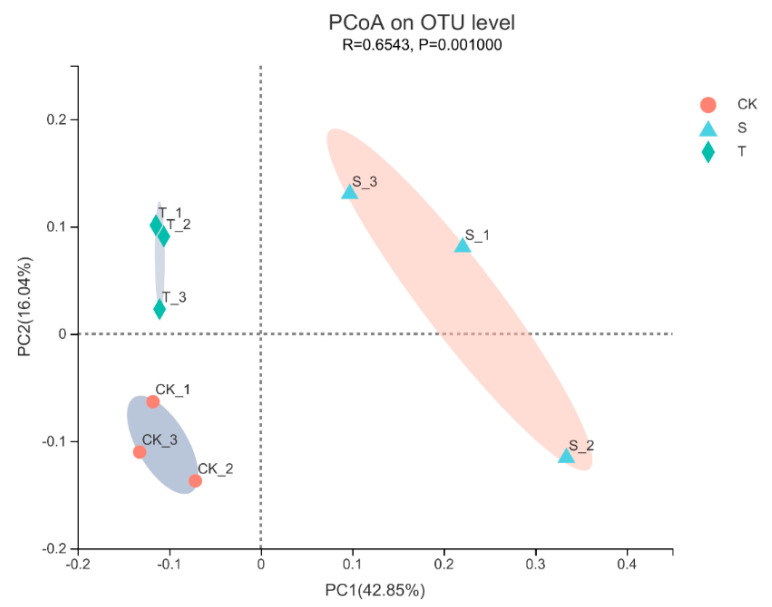
Principal coordinate analysis (PCoA) based on Bray–Curtis dissimilarity at the OTU level between CK: tea plant in monoculture; S: soybean in tea plant/soybean intercropping system; and T: tea plant in tea plant/soybean intercropping system.

Growth and development of plants is intricately linked to microbial abundance and diversity in the soil [18]. Next-generation sequencing technologies provide an efficient way to study complex microbial communities by obtaining operational taxonomic units (OTUs). To assess the role of rhizosphere interactions under tea plant–soybean intercropping, the bacterial community in rhizosphere soil was analyzed using high-throughput sequencing technique of 16S rRNA genes. A total of 437,702 sequences were obtained through the quality filter and chimera check, with an average length of 416 bp. Overall, 4868 operational taxonomic units (OTUs) with a similarity threshold of 97% were found in all samples. Clear asymptotes shown in the rarefaction curves indicated near-complete community sampling (Supplementary Figure S1). To assess the diversity and richness of bacterial community of soils among samples, alpha diversity indices (Sobs, Shannon, Simpson, Ace and Chao) were calculated (Supplementary Table S1). It demonstrated that none of the soil samples differed significantly based on either the OTU or alpha diversity indices, similar to the findings of Huang, et al. [27]. Principal coordinate analysis (PCoA) and 3D-PCoA based on Bray–Curtis dissimilarity showed that rhizosphere soil samples were separated from each other at the OTU level (Figure 4, Supplementary Figure S2). The first principal coordination axis accounted for 42.85% of the total variation, while the second principal coordination axis explained 16.04%. Analysis of similarities (ANOSIM) based on Bray–Curtis also revealed significant differences in composition among different cultivation methods.

### 3.5. Composition of the Bacterial Community in Rhizosphere Soil under Tea Plant-Soybean Intercropping

**Figure 5:**
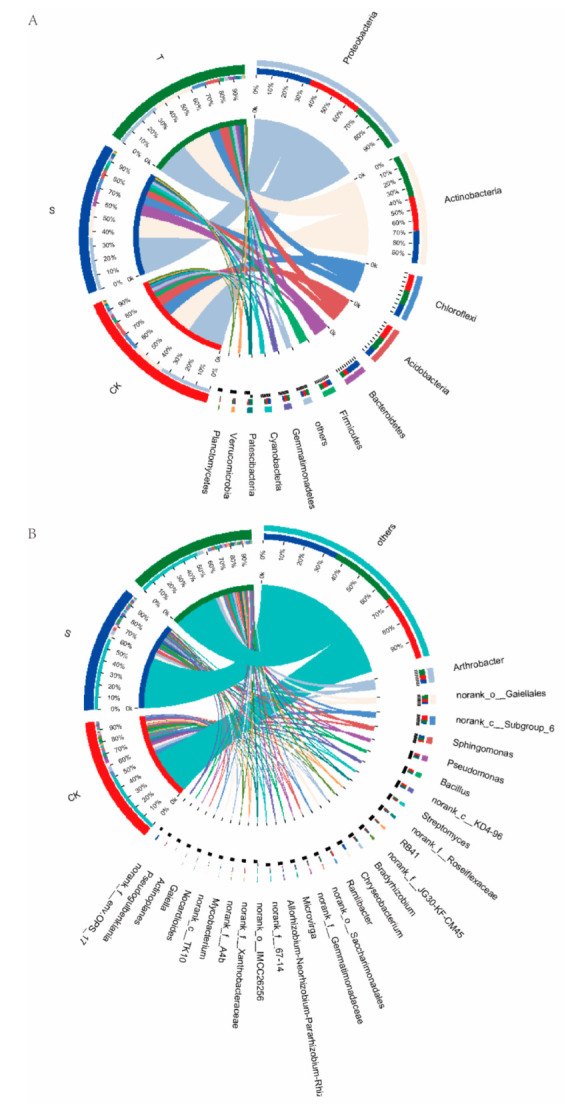
Composition of the bacterial community in tea plantation soils under monoculture and intercropping (soybean–tea plant) systems. Cladogram shows phylogenetic distribution of the most differentially abundant taxa in tea plantation soils under different management at (**A**) phylum and (**B**) genus levels. S, rhizosphere soil of soybean in tea plant/soybean intercropping system; T, rhizosphere soil of tea plants in soybean/tea intercropping; CK, rhizosphere soil of monocultured tea plants.

**Figure 6:**
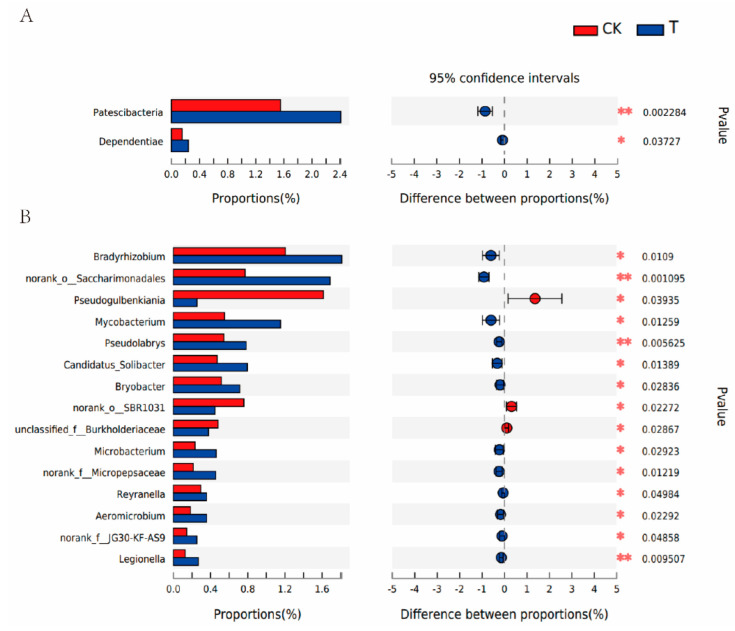
Differential soil bacterial communities significantly differ at (**A**) phylum and (**B**) genus levels. CK: tea monoculture. T: tea/soybean intercropping system. Asterisks represent significant differences (* *p* < 0.05; ** *p* < 0.01).

Intercropped soybean led to compositional changes in the rhizosphere microbiota of the tea plant. Variations in the rhizosphere bacterial community during the legume profuse flowering stage were explored. The relative abundance map of soil bacterial communities (Figure 5) revealed significant differences in the composition of the bacterial community in rhizosphere soil between soybean and tea plants in the different systems. The dominant bacterial communities in the rhizosphere soil of the tea plant were mainly derived from four phyla, Proteobacteria, Actinobacteria, Chloroflexi and Acidobacteria, whereas Bacteroidetes was another dominant phylum in the rhizosphere soil of soybean (Figure 5A), in line with the results of Huang, et al. [27] and Fu, et al. [45]. Proteobacteria dominated the bacterial communities regardless of the practice system and plants, accounting for 36.90%, 33.43%, and 33.15% in monoculture tea plants, soybean, and intercropped tea plants, respectively. Abundance of Actinobacteria was increased by soybean intercropping, whereas abundance of Chloroflexi and Acidobacteria were decreased from 11.94% to 10.41% and 10.40% to 9.48%, respectively, by soybean intercropping. Patescibacteria and Dependentiae were the differential bacteria at the phylum level in the rhizosphere soil of tea plants affected by the soybean intercropping, the abundance of which increased under soybean intercropping compared with monoculture (Figure 6A). Though research on the bacterial phylum Dependentiae is limited, 16S rRNA sequences and metagenomic data revealed that they are widespread across diverse environments [46]. Patescibacteria have the potential to detoxify metals and are considered dominant microorganisms that quickly adapt to extreme environments [47]. Proteobacteria and Actinobacteria are functionally diverse and contribute to decomposition of organic matter, with Gram-negative Proteobacteria found in numerous plant systems [48]. Dai, et al. [49] showed that abundance of Proteobacteria was boosted by increased nutrient availability in the soil. The increased Proteobacteria observed in the intercropped tea plant soil may have been due to the regulated nutrient condition of soybean. This finding is supported by the decreased abundance of Acidobacteria, since Acidobacteria tend to be recruited in nutrient-poor soils [48]. Though research about the bacterial phylum Dependentiae is limited, many 16S rRNA sequences and metagenomic data revealed that they were widespread across diverse environments [46].

Species analysis at the genus level showed significant differences in bacterial communities in rhizosphere soil between monoculture and intercropped systems (Figure 5B). *Arthrobacter* was the most abundant genus in the rhizosphere soil of tea plants, followed by *Gaiellales*, whereas *Chryseobacterium* was most abundant in the soybean rhizosphere. The top 15 differential bacteria at genus level were selected for further analysis. As shown in Figure 6B, intercropped soybean boosted all bacteria in the rhizosphere soil of tea plants, except for *Pseudogulbenkiania*, Burkholderiaceae and *norank-o-*SBR1031. *Pseudogulbenkiania* was the most abundant genus of the differential rhizobacteria in tea plantation under monoculture system, and it significantly decreased from 4.45 ± 0.40% to 2.85 ± 1.02% under tea/soybean intercropping system. *Pseudogulbenkiania* is responsible for denitrification leading to decreased NO_3_^−^ levels in soil [49]. *Bradyrhizobium*, *Saccharimonadales* and *Mycobacterium* were the top three differential bacteria in the rhizosphere soil of tea plants under the intercropping system, with abundance increased by 50.37%, 119.12% and 109.48%, respectively. *Bradyrhizobium* play important roles in promotion of plant growth [50], with monoculture reported to lead to decreased abundance of *Bradyrhizobium* and other beneficial bacteria [23]. *Saccharimonadales* have been shown to be responsible for denitrification, and synergism with *Candidatus Jetteniacan* enable efficient nitrogen conversion [51]. *Mycobacterium* strain Mya-zh01 and *Mycobacterium phlei* were demonstrated to promote plant growth, especially in nutrient-deficient soils [52,53]. These findings indicate that the intercropped soybean could not only directly provide nitrogen resources, but also promote growth of tea plants by inducing activity of related rhizobacteria.

### 3.6. Correlation between Differential Bacteria and Soil Physicochemical Properties

**Figure 7:**
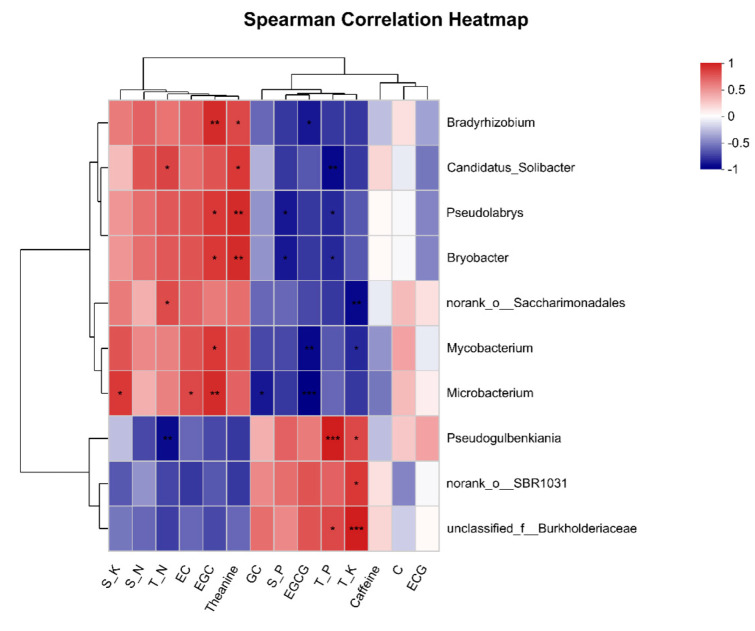
Relationship between top 10 differential rhizosphere bacteria at the genus level and soil physicochemical characteristics, as well as tea quality characteristics. T_N, T_P and T_K represent the content of N, P and K in whole tea plants; S_N, S_P, and S_K represent the content of N, P, and K in the soil. * 0.01 < *p* ≤ 0.05, ** 0.001 < *p* ≤ 0.01, and *** *p* ≤ 0.001.

Soil microorganisms interact extensively with roots and further impact plant mineral nutrition directly by regulating availability and uptake, or indirectly by improving root development [54]. To probe the interaction between tea plant and intercropped soybean, the relationship between differential bacteria, nutrient levels in soil and tea plants, and tea quality characteristics was analyzed (Figure 7). Variance inflation factors (VIF) were selected, and those VIF > 20 were removed from the following correlation analysis. Two types of distinct functional network were found among the top 10 differential rhizosphere bacteria. Specifically, *Pseudogulbenkiania*, norank_o_ SBR1031 and Burkholderiaceae clustered showing a correlation with soil physicochemical and tea quality characteristics, however, other differential bacteria showed the opposite pattern.

Soil properties, including pH and nutrition availability, are important drivers of bacterial community structure, with the correlation between differential bacteria and soil properties, as well as tea quality characteristics, shown in Figure 7. Nitrogen content in tea plants was positively correlated with abundance of *Candidatus Solibacter* and Saccharimonadales, and negatively correlated with *Pseudogulbenkiania*. In addition, phosphorus content in tea plants had a negative correlation with *Candidatus Solibacter, Pseudolabrys* and *Bryobacter,* yet a positive correlation with *Pseudogulbenkiania* and Burkholderiaceae. Potassium in tea plants was negatively related with abundance of norank_o_Saccharimonadales and *Mycobacrerium*, but positively related with *Pseudogulbenkiania,* SBR1031 and Burkholderiaceae. In rhizosphere soil, there was no significant correlation between nitrogen and the top ten differential bacterial genera. Phosphorus was negatively correlated with *Pseudolabrys* and *Bryobacter,* while potassium was positively correlated with abundance of *Microbacterium.* Saccharimonadales are affiliated with the phylum Pastescibacteria, known to be associated with nitrogen cycling. Saccharimonadales are also considered as potential microbial bioindicators of high phosphorus availability and showed synergistic impacts on nitrogen cycling-related genes [55]. However, our study has not shown a distinct positive correlation between P and Saccharimonadales. The influence of Saccharimonadales on P availability in soil for tea plants may have been offset by the strong competition for P utilization in the intercropped soybean (Figure 2). The availability of soil K, which directly affects absorption and utilization of K in plants, is known to be regulated by adjusted soil pH and the content of soil acids [56]. Saccharimonadales has a negative correlation with the content of acids [34], which could explain the negative relationship between K content in tea plants and abundance of Saccharimonadales.

Tea is a nitrogen-loving crop with a preference for ammonium [57]. Li, et al. [58] revealed that *Pseudogulbenkiania* related species have physiological and ecological attributes for nitrogen cycling, mainly involving nitrate reduction. *Pseudogulbenkiania* is responsible for conversion of NO_3_^−^ to NO_2_^−^, NO, and N_2_O, which could explain the significant negative correlation between content of nitrogen in plants and abundance of *Pseudogulbenkiania;* however, there is a lack of information about the effect of *Pseudogulbenkiania* on K and P. *Burkholderiais* perhaps the most varied and ecologically adaptive genus in ecosystems [59]. Shen and Lin [26] found that Burkholderiaceae was a biomarker for cover cropping. Moreover, Burkholderiaceae have recently been shown to dominate in the rhizophere of tea plants, suggesting that they may develop close relationships with tea plants and modulate plant development [60].

Different types of catechins had different correlations with rhizobacteria. *Microbacterium* was negatively correlated with GC, but positively correlated with EC. *Bradyrhizobium*, *Pseudolabrys*, *Bryobacter*, *Mycobacterium* and *Microbacterium* were positively correlated with EGC, while *Bradyrhizobium*, *Mycobacterium* and *Microbacterium* were negatively correlated with EGCG. It was noted that *Mycobacterium* and *Microbacterium* reduced the production of ester type catechins (EGCG) and boosted the production of nonester type catechins (EGC), which is conducive to the tea taste. EGCG is the most abundant catechin component of green tea, accounting for approximately 59% of all catechins. Experimental evidence has shown the importance of the antimicrobial activity of EGCG against *Mycobacterium* [61]. The genus *Microbacterium* belongs to the phylum Actinobacteria, and has the ability to produce the plant growth hormone indoleacetic acid and degrade hydrocarbons [62]. Cordovez, et al. [63] demonstrated that VOCs produced by *Microbacterium* represent a diverse resource to promote plant growth and health, which indirectly support our finding of a positive correlation between *Microbacterium* and tea quality.

There was no significant correlation between the rhizosphere bacteria and C, ECG or caffeine. Theanine is a unique amino acid limited to the *Camellia* genus, except for the basidiomycete mushroom *Xerocomus badius* [64]. In this study, theanine was positively related with *Bradyrhizobium, Candidatus Solibacter, Pseudolabrys* and *Bryobacter* (Figure 7). *Bradyrhizobium* is a N-fixing symbiotic microorganism from soybean, which provides nitrogen resources [65]. Rana, et al. [66] reported that after assimilation, the N was transferred to amino acids, particularly theanine, which could explain the positive correlation between *Bradyrhizobium* and theanine. *Bryobacter* was reported to play a crucial role in the biogeochemical carbon cycle that can utilize various carbon resources [67]. Similarly, *Pseudolabrys* spp. were identified as hydrocarbon degraders and were ubiquitous in hydrocarbon-rich soil [68]. We propose that the reaction products from carbon utilization by these two bacteria could supply basic carbon skeletons for the biosynthesis of amino acids in tea plants.

Soybeans grow vigorously in the profuse flowering stage, which could form a moderate shading effect on young tea plants [69]. The intercropping soybeans with the profuse flowering stage were more beneficial to the growth and the quality of tea plants [9], the variation of bacterial communities during the soybean profuse flowering stage was assessed in this study. In summary, the abundance changes of the *Solibacter*, *Saccharimonadales*, *Pseudogulbenkiania*, *SBR1031,* and *Burkholderiaceae* induced by the intercropped soybean had potential impacts on soil fertility, whereas *Bradyrhizobium*, *Pseudolabry*, *Bryo-bacter*, *Mycobacterium,* and *Microbacterium* with the profuse flowering had potential impacts on tea quality improvement.

## 4. Conclusions

A growing emphasis is being placed on the tea plant/soybean intercropping system in Chinese tea industry. This is in line with the philosophy of achieving carbon neutrality goals. In this study, soybean was selected as the intercropping plant, investigating the impacts of intercropping on soil fertility and tea plant performance and to characterize rhizosphere bacterial community composition during the soybean profuse flowering stage. The results showed that soybean intercropping could boost the uptake of N in tea plants and enhance the growth of young tea shoots. However, interspecific competition and facilitation between intercropped soybean and tea plants were found to coexist, which must be considered when applying this cultivation practice. Tea/soybean intercropping improved tea quality by significantly increasing content of nonester type catechins, total catechins and theanine, while decreasing content of ester type catechins. Analysis of 16S rRNA genes of bacterial communities in tea plantation soils revealed that rhizobacterial community composition and structure were affected by the intercropping system. At the genus level, the relative abundance of beneficial bacteria, such as *Bradyrhizobium*, *Saccha-rimonadales,* and *Mycobacterium*, was increased, whereas the relative abundance of denitrifying bacteria, *Pseudogulbenkiania* was decreased at tea/soybean intercropping. Correlation analysis between differential bacteria and soil properties, as well as tea quality characteristics showed no significant correlation between the catechin or caffeine content and rhizosphere bacteria. Changes in abundance of *Candidatus Solibacter, Saccharimonadales, Pseudogulbenkiania, SBR1031,* and *Burkholderiaceae* suggest potential impacts on soil fertility, whereas *Bradyrhizobium, Pseudolabry, Bryo-bacter, Mycobacterium* and *Microbacterium* induced by the intercropped soybean would have potential impacts on tea quality improvement. Overall, this study improves understanding of the synergistic effect induced by the tea plant–soybean intercropping system and provides a better theoretical reference for the eco-friendly cultivation and management of tea plantations. In a subsequent project, the functional potentiality of the soil bacteria will be verified. Long-term experiments need to be carried out to confirm the effect of soybean intercropping on tea plant growth and tea quality.

## Figures and Tables

**Table 1:** Effect of tea plant/soybean intercropping on the content of N, P, and K in soil and whole tea plants. All data are shown as the mean ± SD (*n* = 3). Data following same letters were not significantly different at *p* < 0.05.

	Treatments	N (mg/kg)	P (mg/kg)	K (mg/kg)
Soil	Tea plant monoculture soil	1010.00 ± 133.17 a	416.18 ± 18.15 a	202.64 ± 1.00 a
	Soy bean monoculture soil	903.33 ± 43.33 a	410.50 ± 48.79 ab	199.96 ± 3.19 a
	Intercropping soil	1190.00 ± 100.17 a	277.79 ± 15.81 b	207.63 ± 1.92 a
Plants	Tea plant monoculture	1656.69 ± 102.38 d	947.50 ± 31.40 c	7967.78 ± 260.30 b
	Soy bean monoculture	2633.46 ± 3.43 b	1707.18 ± 44.54 b	17749.63 ± 456.59 a
	Intercropping tea plant	2160.15 ± 101.96 c	678.81 ± 32.66 d	6823.71 ± 341.90 b
	Intercropping soy bean	3035.21 ± 3.69 a	1996.30 ± 54.51 a	19554.57 ± 943.15 a

## Data Availability

The raw sequencing data were deposited in NCBI Sequence Read Archive (SRA) and the range of 9 SRA accession numbers were SRX17146438–SRX17146446.

## Supplementary Materials

The following supporting information can be downloaded at: https://www.mdpi.com/article/10.3390/microorganisms10112149/s1. Supplementary Figure S1. The rarefaction curve of soil bacterial communities; Supplementary Figure S2. 3D-Principal coordinate analysis (PCoA) based on Bray–Curtis dissimilarity at the out level between (CK: tea plant in monoculture; S: soybean in tea plant/soybean intercropping system; and T: tea plant in tea plant/soybean intercropping system). Supplementary Table S1. Alpha diversity indices soil bacterial communities under intercropping and monoculture system.

## Abbreviations

PGPR: plant growth-promoting rhizobacteria; N-AHL, N-acyl homoserine lactone; SOM, soil organic matter; HPLC, high-performance liquid chromatography; TTC, 2,3,5-Triphenyltetrazolium chloride; CR, competitive ratio; NCR, nitrogen competitive ratio; PCR, phosphorus competitive ratio; KCR, potassium competitive ratio; OTUs, operational taxonomic units; EC, including Epicatechin; EGC, epigallocatechin; C, catechin; ECG, epicatechin gallate; EGCG, epigallocatechin gallate; NIST, national institute of standards and technology; PCoA, principal coordinate analysis; LEfSe, linear discriminant analysis effect size; ANOSIM, analysis of similarities.
