# Supplementary figures and images for: Tea-Soybean Intercropping Improves Tea Quality and Nutrition Uptake by Inducing Changes of Rhizosphere Bacterial Communities
Source: Microorganisms. 2022 Oct 29;10(11):2149. doi: 10.3390/microorganisms10112149 (PMC9697773; doi:10.3390/microorganisms10112149)

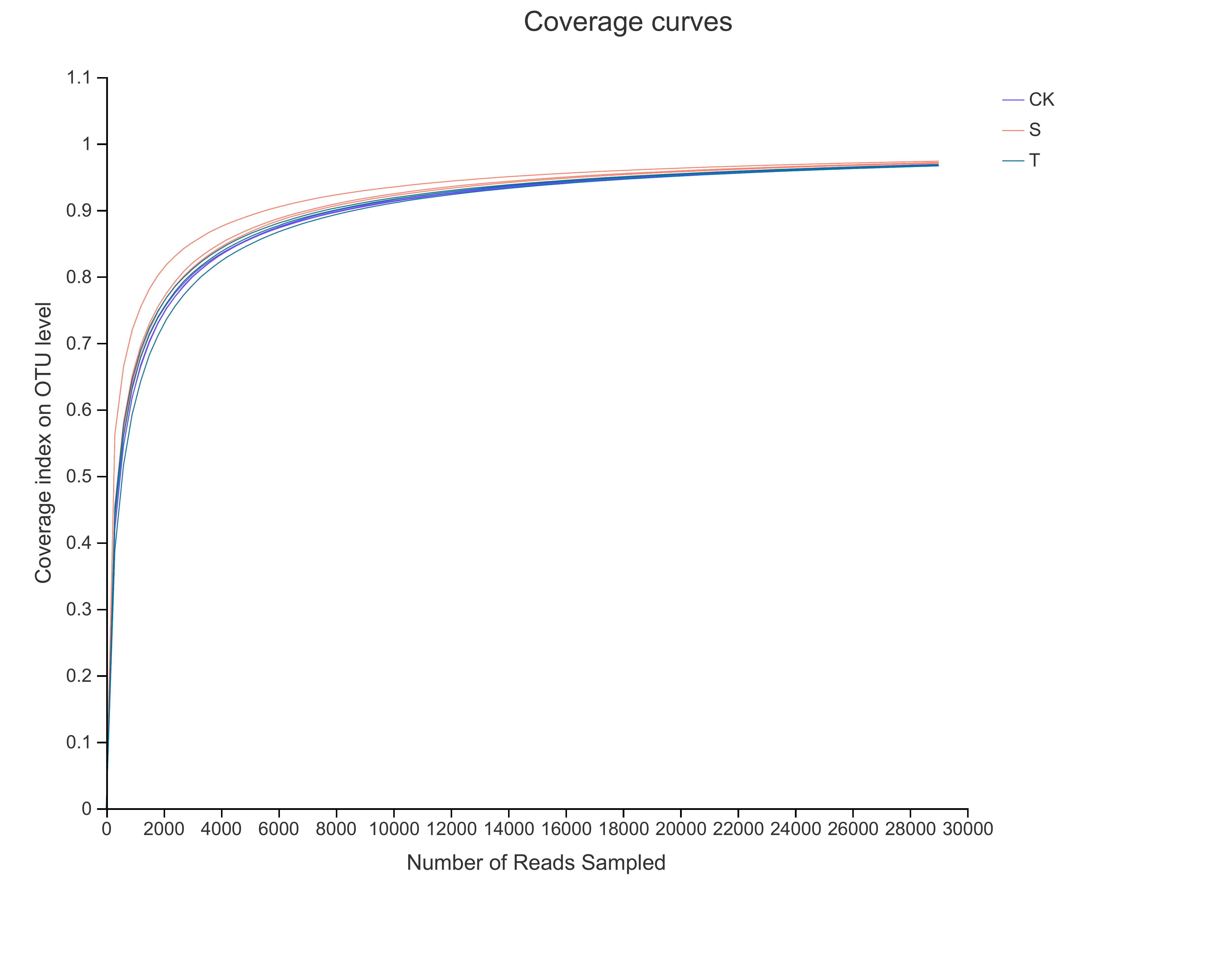

Supplement: Supplementary file 1 [file microorganisms-10-02149-s001.zip › Supplementary Figure S1. The rarefaction curve of soil bacterial communities.png]

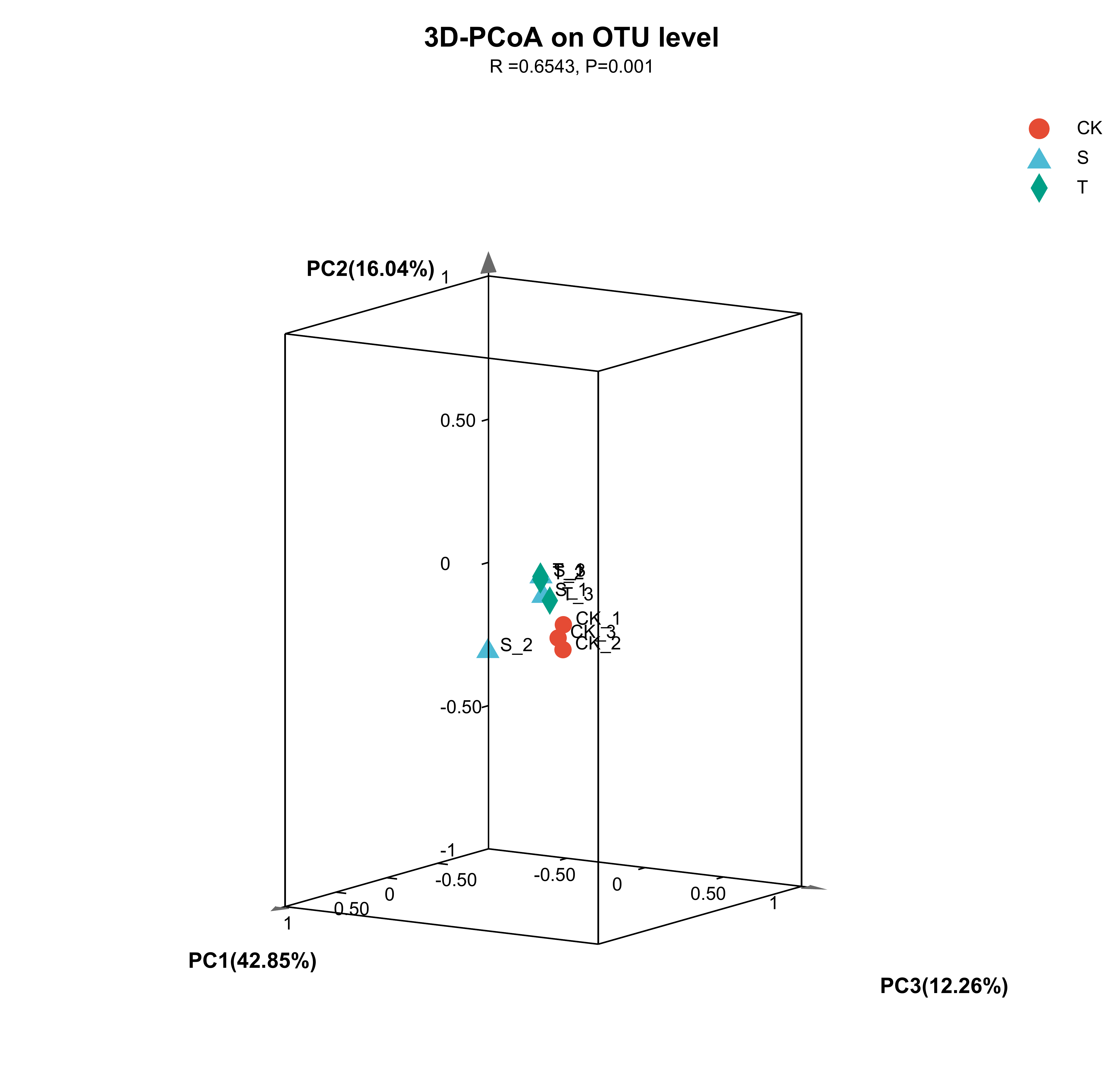

Supplement: Supplementary file 1 [file microorganisms-10-02149-s001.zip › Supplementary Figure S2. 3D-PCOA.png]
